# Effectiveness of the Functional Movement Screen for assessment of injury risk occurrence in football players

**DOI:** 10.5114/biolsport.2022.107482

**Published:** 2021-11-10

**Authors:** Marek Łyp, Marcin Rosiński, Jarosław P. Chmielewski, Małgorzata A. Czarny-Działak, Magdalena Osuch, Daria Urbańska, Tomasz Wójcik, Magdalena Florek-Łuszczki, Iwona A. Stanisławska

**Affiliations:** 1 College of Rehabilitation in Warsaw, Poland; 2 Faculty of Medicine and Health Sciences, Kielce The Jan Kochanowski University, Poland; 3 Uniwersytet Jagielloński – Collegium Medicum w Krakowie, Poland; 4 Institute of Rural Health, Lublin, Poland

**Keywords:** FMS test, Diagnostic tool, Injury, Risk assessment of injury

## Abstract

The aim of the study was to determine whether the Functional Movement Screen (FMS) test carried out among young boys practising football training identifies previous injuries. Sixty-five boys aged 12–13 years, who had regularly practised football in an academy for at least 3 years, were recruited and divided into two groups: an injured group (IG), consisting of players who had experienced at least one injury in the past (n + 25, age 12.32 ± 0.48) and a non-injured group (non-IG), a control group, made up of athletes with no injuries to the musculoskeletal system (n = 40, age 12.25 ± 0.49). Seven FMS tests were used to rate the functional fitness level as a part of the FMS tool. Significant differences between the total scores of the FMS tests (p < 0.001, r = 0.54) were documented. Higher scores in the FMS test were observed in the control group (M = 16.58, SD = 2.04) than in the study group (M = 14.20, ± SD = 1.96). The FMS test is an effective diagnostic tool to identify previous injuries among young football players.

## INTRODUCTION

The Functional Movement Screen (FMS) [4, 5, 6] test compared to other tests assessing fitness is distinguished by an approach focusing on assessing and measuring the asymmetry of the body through the study of basic movement patterns. Among novice raters, the FMS composite score demonstrated moderate to good inter-rater and intra-rater reliability, with acceptable levels of measurement error. The measures of reliability and measurement error were similar for both intra-rater reliability that repeated the assessment of the movement patterns over a 48- to 72-hour period and inter-rater reliability that had 2 raters assess the same movement pattern simultaneously. The inter-rater agreement of the FMS component scores was good to excellent for the push-up, quadruped, shoulder mobility, straight leg raise, squat, hurdle, and lunge. Referring to the data of the US National Electronic Injury Surveillance (NEISS), it can be noted that damage to the locomotor system in the group of children training up to 12 years of age in the United States occurs with a frequency of 2 cases per 1000 [17]. In children from 12 to 15 years of age, the rates were 4–7.6 per 1000 people [8, 16, 30]. Its utilization among football players, who in practice are most often tested by fitness tests, focused on typical technical and tactical skills, is particularly valuable, especially in the context of prevention and prophylaxis of injury. The FMS test helps to identify the source of a person’s movement problems, as inadequate levels of movement efficiency that may depend on many factors, including strength, endurance, neuro-muscular coordination, speed, and agility. Corrective exercise programmes included in the FMS test and their progressions allow for improvement in a specific basic movement pattern. Together with the achievement of this goal, when the football player begins to function more effectively, his performance is significantly improved, and this situation reduces the risk of injury during sport activity. The history of sports injuries should always be taken into account when performing the FMS test because one of the main predictors of injuries of the training individual is the occurrence of any previous injuries during the training sessions and competitions [13, 14]. The authors have presented research that aims to evaluate whether the injuries sustained in the past have any influence on the score of the FMS test.

The aim of the study was to evaluate whether the FMS test is an effective diagnostic tool to identify previous injuries in 12–13-year-old boys taking part in football training.

## MATERIALS AND METHODS

The FMS tests took place in a closed room in order to exclude the influence of external factors. The study included 65 boys aged 12–13 who had been regularly (at least three times a week) practising football. The 65 boys were divided into two groups: the study group and the control group. The study group consisted of players who, before the study began, had sustained injuries of at least one locomotor system during their training or a match. The control group consisted of players who had never experienced any injury. An injury was defined in this case as an incident during a game or a training session which simultaneously meets 3 conditions: (1) medical assistance was needed, (2) which rendered the player unable to participate in the rest of the activity and (3) he was absent from any subsequent physical activity for at least 4 weeks. There were 40 players aged 12.25 (± 0.49) in the control group and 25 players aged 12.32 (± 0.48) in the study group. Both groups had their anthropometric features measured: height, weight, and BMI (Table 1). In both groups, a qualitative assessment of the movement was performed based on the FMS test which consists of 7 exercises: 1. deep squat, 2. hurdle step, 3. in-line lunge, 4. shoulder mobility, 5. active straight leg raise, 6. trunk stability push-up, and 7. rotational stability. Each of the study participants performed these 7 exercises, assessed on a scale of 0 to 3 points, where 0 was given in the event of pain. The maximum number of points that could be obtained by the participant was 21 (Table 2).

**TABLE 1:** Comparison of anthropometric features of the control and study group.

	Uninjured		Injured		U Mann–Whitney test	
	M	SD	M	SD	P	r
Age	12.25	0.49	12.32	0.48	0.602	0.06
Height [cm]	152.90	8.64	156.52	9.95	0.224	0.15
Weight [kg]	40.58	6.71	44.20	11.41	0.388	0.11
BMI	17.24	1.45	17.76	2.89	0.845	0.02

M – average; SD – standard deviation; p – level of significance; r – strength of the effect

**TABLE 2:** The differences in seven movement patterns (FMS tests) between injured and non-injured young soccer players

	Uninjured		Injured		U Mann–Whitney test	
	M	SD	M	SD	P	r
Test 1	2.45	0.60	1.72	0.68	< 0.001	0.50
Test 2	2.48	0.60	2.36	0.70	0.566	0.07
Test 3	2.55	0.64	2.04	0.61	0.001	0.41
Test 4	2.63	0.59	2.48	0.77	0.577	0.07
Test 5	2.30	0.56	1.92	0.76	0.036	0.26
Test 6	2.18	0.78	1.80	0.71	0.055	0.24
Test 7	1.95	0.55	1.84	0.62	0.439	0.10
FMS result	16.58	2.04	14.20	1.96	<0.00	0.54

M – average; SD – standard deviation; p – level of significance, r – strength of the effect

All calculations were performed using STATISTICA ver. 13.3. The chi-square test was used as a non-parametric test in evaluating the differences between the two variables on the nominal scale. This test was performed to compare the ordinal variables. The degrees of freedom (df) and the levels of statistical significance (p) were determined. The sensitivity and specificity for individual thresholds of the scale division were also determined, which gives us information about the ability of the test to detect the tested feature or its absence, respectively.

Non-parametric correlation methods were used to detect any possible relationship between the two variables and to estimate the strength and the statistical significance of this relationship. Cramer’s V coefficient based on the chi-square test for 2 x 2 contingency tables and the j coefficient for tables with larger dimensions were used as a measure of relationship. In the case of variables on the ordinal and nominal scales, we are only able to determine the strength of the relationship (effect) r and the statistical significance p of the relationship in the study. Spearman’s r coefficient was used as a measure of the explained variability. A scale was adopted for the directly proportional strength of the effect r, i.e., r > 0: r < 0.3 being a weak relationship, r < 0.5 being a medium relationship, and r < 0.5 being a strong relationship. The analysis assumed the level of significance of p < 0.05. The significance was distinguished as follows: p < 0.05; p < 0.01; p < 0.001.

### Ethics

Experiments reported in the manuscript were performed in accordance with the ethical standards of the Helsinki Declaration. The participants signed an informed consent form. Approval of the Bioethics Committee at the College of Rehabilitation in Warsaw, No. 23/2017 from 22^nd^ July 2017, was obtained.

## RESULTS

The relationship between players’ injury occurrence and anthropometric features was assessed at the beginning. No significant differences between the study group and the control groups were found in terms of age (p = 0.602, r = 0.06); height (p = 0.224, r = 0.015); weight (p = 0.388, r = 0.11), and BMI (p = 0.845, r = 0.02) (Table 1).

Evaluation of the relationship between players’ injury occurrence and results of the FMS test was one of the most important research issues in this study. A comparison of average results of the FMS test of the control and the study group was performed (Table 2). Statistically significant differences were found between the groups in terms of the result in the *first* (p < 0.001; r = 0.50) and the *third tests* (p < 0.01, r = 0.41). The rating of the performance of a deep squat in the control group was significantly higher (M = 2.45, SD = 0.60) than in the study group (M = 1.72, SD = 0.68). Based on the effect size (r = 0.50), the difference between the groups in the test was significant. Moreover, a squat in a lunge was rated higher in the control group (M = 2.55, SD = 0.64) than in the study group (M = 2.04, SD = 0.61). Based on the effect size (r = 0.41), the difference between the groups in the test was moderate. No differences were observed between the control and the study groups in relation to the rating in the second and the fourth test (p = 0.566, r = 0.07 and p = 0.577, r = 0.07, respectively). The movement of the lower limb over the fence was rated similarly in the control group (M = 2.48, SD = 0.60) and in the study group (M = 2.36, SD = 0.70). Moreover, the mobility of the shoulder girdle was similarly rated in the control group (M = 2.63, SD = 0.59) and in the study group (M = 2.48, SD = 0.77). Differences between the groups were observed in the fifth and sixth tests (p < 0.05, r = 0.26 and p = 0.055, r = 0.24, respectively) (borderline significant result). The rating of the performance of active elevation of the straight lower limb in the control group was significantly higher (M = 2.30, S = 0.56) than in the study group (M = 1.92, SD = 0.76). However, based on effect size (r = 0.26), it can be assessed that the difference was insignificant. The rating of the performance of deflection of the arms in the support was also rated higher in the control group (M = 2.18, SD = 0.78) than in the study group (M = 1.80, SD = 0.71). No difference was observed in the seventh test (p = 0.439, r = 0.10). The rotational stability of the trunk was similarly ranked in the control group (M = 1.95, SD = 0.55) and in the study group (M = 1.84, SD = 0.62). A difference in the combined FMS test result was found between the groups (p < 0.001, r = 0.54). The control group was rated higher in the FMS test (M = 16.58, SD = 2.04) than the study group containing injured players (M = 14.20, SD = 1.96). Based on the effect size, r = 0.54, the difference in combined results of the FMS test was significant (Table 2).

In the part concerning the relationship between the football players’ injury occurrence and a number of functional asymmetries determined in FMS tests, it was evaluated whether the number of functional asymmetries correlated with the risk of injury occurrence. The analysis conducted using the Mann–Whitney U test revealed a statistically significant difference between the players of both groups in relation to the number of asymmetries in the conducted tests (p < 0.01, r = 0.34). Based on the effect size (r = 0.34), the difference was moderate (Table 3).

**TABLE 3:** Difference between the average test result in the injured and non-injured groups in relation to number of asymmetries in FMS test

	Uninjured		Injured		U Mann–Whitney test	
	M	SD	M	SD	P	r
Quantity of asymmetries	0.95	1.04	1.64	1.08	0.007	0.34

M – average; SD – standard deviation; p – level of significance, r – Effect size

One of the priorities is the evaluation of the relationship between the scored FMS test and the risk of injury occurrence that is term a determination of the FMS test result threshold indicating increased risk of injury occurrence. For the 14 points, a threshold of ≤ 14 points and > 14 points was determined. For this purpose, a series of analyses using the chi-square test were performed (Table 4).

**TABLE 4:** The results of the Chi-square test of independence for the relationship between FMS score threshold and the occurrence of injuries.

Points	χ^2^	df	p	φ	Sensitivity	Specificity
12	1.06	1	0.304	0.13	0.08	0.98
13	6.57	1	0.010	0.32	0.32	0.93
14	18.66	1	< 0.001	0.54	0.64	0.88
15	17.96	1	< 0.001	0.53	0.76	0.78
16	7.64	1	0.006	0.34	0.84	0.50
17	13.27	1	< 0.001	0.45	1.00	0.40

χ^2^ – chi-square test; df – number of degrees of freedom; p – statistical significance; φ – strength of the effect.

It was demonstrated that for the majority of tested threshold points there was a relationship between a particular threshold point assumed in the FMS test and the injury occurrence. Based on the coefficient of the strength of the effect φ, the most significant correlation could be observed at the threshold of 14 points (χ^2^(1) = 18.66, p < 0.001, φ = 0.54) and 15 points (χ^2^(1) = 17.96, p < 0.001, φ = 0.53).

Variation of sensitivity and specificity for particular thresholds was also evaluated. The best results were obtained for the threshold of 15 points. Players who scored higher than 15 points were classified as not injured with the correctness of 78% and players who scored 15 or below were classified as injured with the correctness of 76%.

In the study an attempt was also made to evaluate the relationship between the type of measurement of the parameter and the risk of injury occurrence. The results of the FMS test were divided into three groups in terms of the type of the parameter: tests of movement quality (first, second, and third test), tests of mobility (fourth and fifth test), and tests of stability (sixth and seventh test) (Table 5).

**TABLE 5:** The differences between risk of injury occurrence and type of the test.

	Non-injured		Injured		U Mann–Whitney test	
	M	SD	M	SD	P	r
Tests of movement quality	2.49	0.43	2.04	0.47	0.001	0.50
Tests of mobility	2.46	0.46	2.20	0.43	0.025	0.28
Tests of stability	2.06	0.52	1.82	0.52	0.058	0.24

M – average score in FMS; SD – standard deviation; p – level of significance, r – Effect size

The analysis performed using the Mann–Whitney U test demonstrated significant differences between the control and the study groups in terms of tests of movement quality (p < 0.001, r = 0.50), tests of mobility (p < 0.05, r = 0.28), and tests of stability (p = 0.058, r = 0.24). Lower scores were observed in relation to tests of movement (M = 2.04, SD = 0.47 vs. M = 2.49, SD = 0.43), tests of mobility (M = 2.20; SD = 0.43 vs. M = 2.46; SD = 0.46), and tests of stability (M = 1.82, SD = 0.52 vs. M = 2.06, SD = 0.52). Based on the effect size coefficient, the most significant differences were observed in tests of movement quality (r = 0.50).

## DISCUSSION

The most important finding was that the FMS test carried out among young boys performing football training identifies previous injuries. This subject is relatively often mentioned in articles related to medicine of sport. During a study of articles related to dependence “FMS score – injury”, a distinction between the types of the study should be made at the beginning as the study can be retro- or prospective. McCunn et al. reported that sixteen articles could be found related to prospective studies on the relationship between the final FMS test results and injury occurrence [20]. Eight of them indicated a significant “FMS score – injury” correlation [2, 3, 9, 13–15, 18, 26]. In the case of these studies, it was demonstrated that, in the vast majority (six out of eight studies), the risk of injury occurrence was higher for those players who obtained a total score of 14 points or less, but the correlation varied. It was influenced by several factors, i.e. number of players participating in the study, length of the observation time, and type of sport practised, which could have had a significant impact on the inconsistency of dependency level between injuries and the FMS test result, whereas the second group of eight studies [1, 7, 10, 21, 22, 28, 29, 31] did not demonstrate any dependence between the FMS test score and experienced injury. However, it should be noted that three of the studies [7, 21, 31] were underpowered due to the low number of participants and scheme of collecting data of injury and seem to be inconclusive, which can explain the lack of correlation and requires caution when evaluating the usefulness of the FMS test for detection of increased risk of injury occurrence.

The second type of correlation “FMS test score – injury” is retrospective. This approach is a result of the assumption that past injuries are the best indication of risk of another injury occurrence [23, 24]. An additional methodological matter in this case, differentiating retrospective studies, is the necessity of medical history interview and classification of information on health of a particular player. It needs to be mentioned that there is no perfect method of reporting injuries as even medical documentation is not a reliable instrument [12, 25, 27]. It seems that the most optimal method is gathering information from the tested person with whom the researchers conduct a detailed interview for detailed identification of any potential injuries. This approach ensures a moderate to high credibility level [11, 19]. This study adopted a similar approach. Because of the young age of tested players, the questionnaires concerning past injuries and general health condition were filled in based on the medical documentation and consulted with parents.

Another issue related to the relationship between injuries and the final FMS test result is the determination of a threshold point below which the risk of injury occurrence increases significantly. The first researchers who presented a study on the “FMS test score – injury” correlation were Kiesel et al., who in the span of 4.5 months tested 46 American football players [14]. Based on their observations, they determined that the greatest sensitivity and specificity of the FMS test were for the threshold of 14 points. Specificity and sensitivity of the diagnostic test are the values describing the ability of the test to correctly detect the examined feature (sensitivity) and detection of lack of the feature (specificity). These terms are being used in studies, including in medical tests used in medicine. The closer both values are to 1, the better the particular test is as a diagnostic instrument used in determining the presence of a feature or lack thereof. The specificity of 100% means that the healthy participants will be indeed classified as healthy. Furthermore, the sensitivity of 100% means that all the sick participants or the participants with a tested disability will also be correctly classified. In the case of the FMS test, the sensitivity is the ability of classification of participants with past injuries and the specificity is the ability of classification of participants without injuries. While determining the threshold of the increase of injury at the level of 14 points, Kiesel et al. achieved specificity of 0.91 and sensitivity of 0.54 [14]. It means that half of the injured players were detected by the FMS test only while using the threshold of 14 points. Nevertheless, the threshold determined by Kiesel et al. is very frequently quoted in articles and other studies focused on the correlation between the final FMS result and the injury occurrence, and it is treated as a reference in the analysis of the results [14]. In this study, the results were analysed not only in terms of the aforementioned threshold of 14 points, but also in relations to other thresholds to determine the optimal level for which sensitivity and specificity would be the best, i.e. that which would be the best threshold allowing one to determine the risk of injury occurrence. The analysis demonstrated that for almost each of the tested thresholds of the total FMS test score there was a correlation between a particular threshold and an injury occurrence. The highest score of the strength of the effect φ was observed for the threshold of 14 and 15 points (0.54 and 0.53; p < 0.001, respectively). The variation of sensitivity and specificity for particular thresholds was also checked. It was assessed that, in relation to these features, the best threshold is 15, as sensitivity was 0.76 and specificity was 0.78. It means that for the threshold of 15 points, 78% of the players were correctly classified as uninjured whereas 76% of the players were classified as individuals who have experienced injury. Interestingly, in the study for the threshold of 16 points, the sensitivity of 0.84 and specificity of 0.50 were obtained, i.e. values similar to Kiesel et al. using a factor of 14 [14]. Possibly, the variation resulted from the differences between the control and the study groups, which differ not only in age (adults – children) but also in the type of sport (American football – football). This makes finding a common denominator for meaningful and objective comparisons extremely difficult.

## CONCLUSIONS

The Functional Movement Screen (FMS) is an effective diagnostic tool to identify previous injuries among young football players.

The largest differences were observed in the first (p < 0.001, r = 0.50) and the third test (p < 0.01; r = 0.41). In the control group, the assessment of the performance of deep squat and in-line lunge was significantly higher than in the study group (M = 2.45, SD = 0.60 vs. M = 1.72, SD = 0.68 and M = 2.55, SD = 0.64 vs. M = 2.04, SD = 0.61).

The threshold in the FMS test below which the risk of injury occurrence increases is 15.

Anthropometric features such as age, height and weight did not influence occurrence of players’ injuries.
